# Noninvasive inset-integrated meta-atom for achieving single-layer metasurface simultaneously with coded microwave reflectivity and digitalized infrared emissivity

**DOI:** 10.1515/nanoph-2024-0098

**Published:** 2024-05-16

**Authors:** Hui-Ting Sun, Jun Wang, Rui-Chao Zhu, Zun-Tian Chu, Xin-Min Fu, Yu-Xiang Jia, Yi-Na Cui, Ya-Juan Han, Tian-Shuo Qiu, Sai Sui, Jia-Fu Wang, Shao-Bo Qu

**Affiliations:** 66488 Air Force Engineering University, Xian, 710051, China; Shaanxi Key Laboratory of Artificially-Structured Functional Materials and Devices, Xian, 710051, China; Suzhou Laboratory, Suzhou, 215000, China

**Keywords:** noninvasive inset-integration, coded microwave reflectivity, digitalized infrared camouflage, single-layer metasurface

## Abstract

With the rapid improvement of equipment integration technology, multi-spectrum detectors are integrated into compact volumes and widely used for object detection. Confront with this challenge, it is essential to propose a strategy to design a single-layer metasurface with multi-spectrum responses in microwave and infrared ranges. In this work, we proposed a method of designing meta-atoms, which is capable of achieving functional electromagnetic response at microwave and infrared individually. As a demonstration, a metasurface with four different occupation ratios and coding permutation features is designed, fabricated, and tested. In the microwave band, the pixel meta-atom is designed to realize highly efficient cross-polarization conversion between 5.0 and 10.0 GHz, which shows the metasurface can behave as ultra-low Radar Cross Section (RCS) reflectors in the working band; In the infrared band, different occupation ratio of meta-atoms are designed to realize the infrared emissivity from 0.60 to 0.80 in 3–14 μm, which can be used to exhibit digital infrared camouflage pattern. This work promotes the ability to use single-layer design to achieve digital infrared camouflage and microwave RCS reduction simultaneously. The one-layer design is simple in geometry, simplified in process, low cost in economy, and large scale in fabrication, which can promote practical use in compatible microwave stealth and infrared camouflage.

## 1 Introduction

With the development of modern science and technology, detection techniques are more and more diversified, and it is difficult for single-frequency stealth materials to adapt to the complex and diverse detection environment [1], [2]. Radar and infrared bands are the main bands for detection, thus the realization of radar stealth and infrared camouflage have always been the main methods to improve the survival rate of equipments [3], [4], [5]. Therefore, the compatibility of radar stealth and infrared camouflage is the development direction of stealth materials. According to Kirchhoff’s law, in thermal equilibrium state, for a given temperature and wavelength, the emissivity and absorptivity of an object are numerically equal. For opaque materials, a low emission rate means a low absorption rate and high electromagnetic reflection. Note that radar stealth requires a high absorption rate and low reflectivity, thus microwave radar stealth and infrared stealth are contradictory in implementation [6]. Therefore, it is difficult for traditional materials to have both high microwave absorption and infrared camouflage. In earlier studies, semiconductors and other compounds were mixed to control the reflection and absorption of the coating to achieve infrared and microwave compatibility [2], [3], [4]. Moreover, some researchers have proposed the use of one-dimensional double hetero-structure photonic crystals as the infrared low-emission layer, which makes no difference to the microwave frequency band [7], [8]. This method can be applied to stealth-compatible materials of infrared microwave radar. Although these methods can achieve compatibility in radar and infrared bands simultaneously, the manufacturing process is complex and high cost, which is not beneficial in large-scale engineering applications. Moreover, in the motley background environment, the realization of infrared camouflage should not be limited to low or high infrared emissivity [9]. It is better to pay attention to the gradient change of infrared emissivity to adapt to the complex infrared background, where low infrared radiation and high infrared radiation coexist, such as forests, bushes, and densely populated construction areas. However, traditional infrared camouflage materials make it difficult to achieve the gradient change of infrared emissivity [10], [11]. As a new type of artificial surface, the metasurface is a periodic or quasi-periodic meta-atom array, which has been widely used in infrared camouflage and microwave stealth design recently [12]. The introduction of the metasurfaces provides a new idea for multi-spectrum compatible design [9], [13], [14], [15]. By superimposing different functional surfaces, multiple functions within different frequency bands can be implemented in one meta-atom [10]. Specifically, a common design is established in compounding a frequency selective surface (FSS) with a gradient occupation ratio at the top layer and other absorption or scattering layers at the bottom [5], [16]. However, the multi-layer design structure constructed via this method inevitably leads to bulky meta-devices, interlayer coupling, and low integration. Under such circumstances, the design of a single-layer metasurface with compatible functions in infrared and microwave bands has become the solution recipe of urgent current.

This paper proposes a noninvasive inset-integrated single-layer multi-functional metasurface (SMM) to realize the digitally camouflaged infrared emissivity by changing the occupation ratio, as well as to generate the highly efficient cross-polarization conversion in the microwave band simultaneously [17]. The noninvasive unit means that the structure is inset without aggression and destruction, referring to the addition of metal patches to the original resonant ring to regulate the infrared emissivity, which do not weaken the microwave performance of the meta-atom.

To design the microwave polarization conversion of the metasurface, the coding arrangement is implemented via two types of meta-atoms with phase difference *π*, which can realize the reduction of RCS through scattering offset. With the assistance of multiple plasmon resonances theory, the origins of polarization conversion at different resonant frequencies are analyzed, and based on that, the broadband polarization conversion is achieved. Meanwhile, the parameters of meta-atom structure correlated to polarization conversion are further optimized to enhance the conversion efficiency. The digital camouflage metasurface proposed in this paper can be better integrated with the complex infrared backgrounds to achieve the effect of camouflage. Moreover, the infrared digital camouflage metasurface proposed in this paper provides a higher degree of freedom for designing the infrared radiation. Without affecting the microwave performance, the camouflage can be customized according to different infrared backgrounds.

## 2 Design and analysis

### 2.1 Design and analysis of the polarization rotator

**Figure 1:**
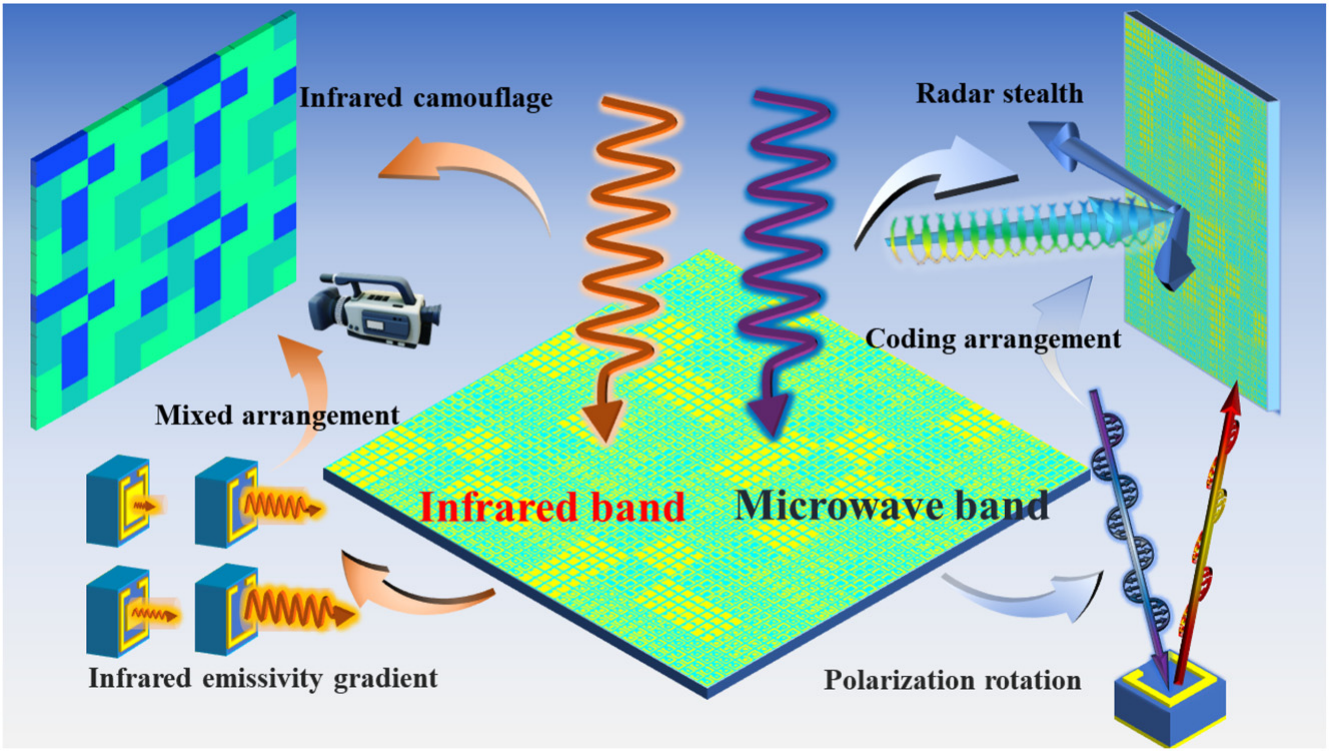
The schematic framework of the multi-functional metasurface.

As shown in Figure 1, the initial polarization rotator has a large area of nonmetallic area, the infrared emissivity of the surface can be locally changed by filling the metal in these areas. The special advantage to use a polarization rotator is that it can provide a lot of infrared devisable space for the independent functional design at microwaves and infrared bands. Therefore, it is capable to achieve RCS reduction in microwave while assemble infrared camouflage patterns in the same functional layer. This additional design can yield digital infrared camouflage patterns on demand and does not affect the original polarization conversion effect. After the verification of simulation and experiments, the results show that the metasurface enables RCS reduction at 5.0–10.0 GHz, as well as achieving the infrared emissivity coverage from 0.47 to 0.80 in the infrared band between 3 and 14 μm. This design method provides a new route to achieve highly compatible infrared camouflage and radar stealth. The single-layer design extremely simplifies the fabrication process, which promotes large-scale samples with rapid time and low-cost.

**Figure 2:**
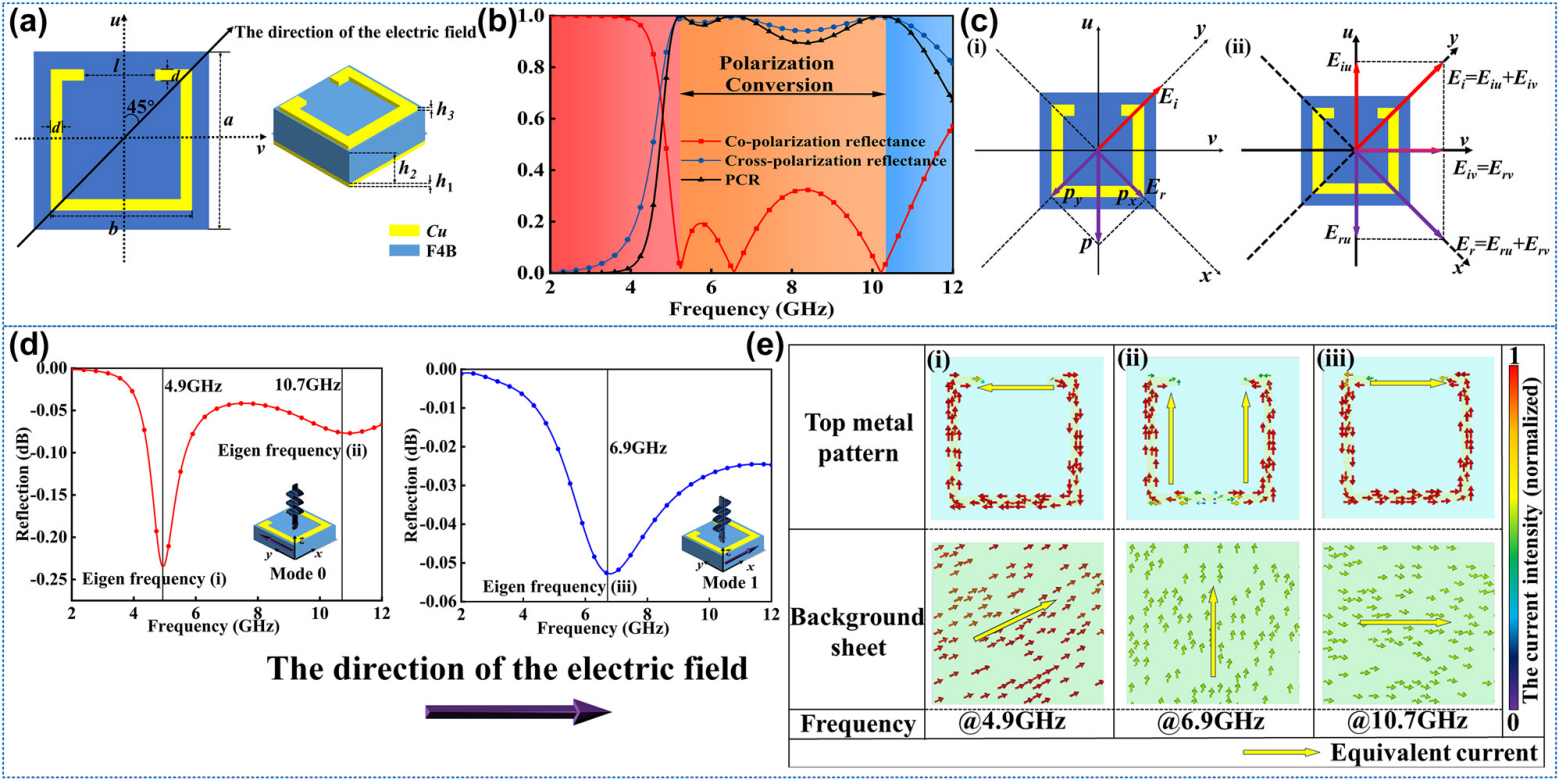
The structure design and mechanism analysis of polarization rotator. (a) The geometric parameters of the rotator. (b) The performance on the polarization conversion of the rotator. (c) The intuitive explanation of the *y*-polarized incident wave rotated to the *x*-polarized reflection wave. (d) The conditions of two typical modes for the polarization conversion and the eigen frequencies of mode 0 and mode 1. (e) The surface current distributions at different frequencies correspond to eigenmodes.

A broadband polarization rotator is designed at first to achieve cross-polarization reflectance in microwave as shown in Figure 2(a). The rotator is composed of three parts: the bottom is copper, the middle substrate is polytetrafluoroethylene (PTFE), and the top is a square copper ring with a gap. The model in the Computer Simulation Technology (CST) Microwave Studio is F4B (with a dielectric constant of 2.65 and a loss tangent of 0.001). To strengthen the coupling of interfacing meta-atoms in the simulation environment, the incident direction of the electric field is set up to 45° and the intersection angle between the open square ring and *y*-axis is 45°. The geometric parameters of the rotator are as follows: *a* = 8 mm, *b* = 6 mm, *d* = 0.4 mm, *l* = 3 mm, *h*_1_ = 0.018 mm, *h*_2_ = 4.5 mm, *h*_3_ = 0.018 mm. Therefore, the rotator can be regarded as a single-anisotropic homogeneous metamaterial layer (of a thickness *h*_1_ + *h*_2_ + *h*_3_) with a dispersive relative permeability tensor and a relative permittivity, put on top of a metallic ground sheet.

Then, the basic rotator is simulated in the CST Microwave Studio, and the performance of the polarization conversion is shown in Figure 2(b). Through the results, the rotator resonates at 4.9 GHz, 6.9 GHz, and 10.7 GHz. The three continuous resonant frequencies are connected into a broadband polarization conversion in the 5.00–10.00 GHz range. Due to the equivalence of the co-polarization and cross-polarization, the reflectance of *r*_*xx*_ = *r*_*yy*_ and *r*_*yx*_ = *r*_*xy*_. The polarization conversion ratio (PCR) is defined as Equation (1) [17], [18]:

(1) $$\text{PCR}=\frac{r_{xy}^{2}}{r_{xx}^{2}+r_{xy}^{2}}=\frac{r_{yx}^{2}}{r_{yy}^{2}+r_{yx}^{2}}$$

where *r*_*xx*_ = *r*_*yy*_ represents co-polarization and *r*_*xy*_ = *r*_*yx*_ indicates cross-polarization, respectively [18], [19]. According to Equation (1), the PCR is calculated and shown in Figure 2(b) as well. The PCR is nearly 90 % from 5.0 GHz to 10.0 GHz. To explain the process of polarization conversion, we take *y*-polarization as input and *x*-polarization as output for example, and the *u*- and *v*-coordinate system is introduced [20], [21], [22]. The *u*-axis is along a 45° direction concerning the *y*-axis and perpendicular to the *v*-axis [17], [23], as shown in Figure 2(c). Then we consider that the incident electromagnetic (EM) wave is polarized along the *y*-axis. The electric field can be decomposed into two orthogonal components (directions $\vec{u}$ and $\vec{v}$ ). Hence, the electric field of the incident EM wave can be expressed as Equation (2) [17]:

(2) $$\vec{E_{i}}=\vec{u}E_{ui}{\text{e}}^{\text{i}\varphi_{iu}}+\vec{v}E_{vi}{\text{e}}^{\text{i}\varphi_{iv}}$$

where $\vec{u}$ and $\vec{v}$ are the meta-atom vectors in the *u*- and *v*-axes, respectively. The electric field of the reflected wave can be written as Equation (3) [17]:

(3) $$\vec{E_{r}}=\vec{u}E_{ur}{\text{e}}^{\text{i}\varphi_{ru}}+\vec{v}E_{vr}{\text{e}}^{\text{i}\varphi_{rv}}$$

in which *ru* and *rv* are the reflection coefficients along the *u*- and *v*-axes, respectively. When the EM wave is incidented into the surface, the electrons motivated by the waves will move along the copper pattern, and the equivalent direction is the *u*-axes, which means the other direction *v*-axes will not affect the performance of the EM waves (be equivalent to the perfect electric conductor), so the Δ*φ* = 0 and *ru* = *rv*. However, in the *u*-axes directions, because of the resonance oscillation between the surface current and bottom current, the phase of the reflected waves will change, and when Δ*φ* = *π*, the field synthesized by *E*_*ru*_ and *E*_*rv*_ will be changed to the *x*-direction. Therefore, the direction of reflective polarization is rotated by 90° [24], [25], [26], [27]. Take the resonance (i) for example. Generally, *μ*_*v*_ and *μ*_*u*_ are usually not large in most cases. Thus, EM waves can interact directly with the metal floor, resulting in out-of-phase reflection. However, at magnetic resonance 4.9 GHz, *μ*_*u*_ can become very large to behave as a high impedance surface. While *μ*_*v*_ is still not large. *μ*_*u*_ and *μ*_*v*_ are different due to the anisotropic properties of the metasurface. When the *y*-polarized incidence EM wave impinges on the metasurface, it has two orthogonal equal components due to an angle of 45° designed between the *y* and *v* axes, which means *E*_*ui*_ = *E*_*vi*_. Reflective components *E*_*vr*_ *E*_*ur*_ are generated by out-phase and in-phase reflections at resonance 4.9 GHz. That means *E*_*vr*_ has a 180 phase difference concerning *E*_*vi*_ after reflected by the metasurface, while *E*_*ur*_ has the same phase as *E*_*ui*_. Hence, the *y*-polarized incident wave is converted to an *x*-polarized reflected wave.

After explaining the principle of the polarization conversion, Figure 2(d) gives the two cases of *u*-polarized and *v*-polarized, and the co-polarization refection under normal incident corresponding to two cases are found as shown in Figure 2(d) as well. In the *u*-direction, there are 2 modes at 4.9 GHz and 10.7 GHz, respectively; in the *v*-direction, there is one mode at 6.9 GHz. In these modes, the resonances between the upper metal and the EM waves are strongest, which leads to the phase difference of the reflective waves.

To analyze the different modes, the surface current is observed by the field monitor in the CST. The distributions and directions of the current in the front and bottom are shown in the Figure 2(e). From mode (i), the equivalent current on the front is from one side of the gap on the ring to the other, and the current on the bottom is anti-parallel to the front. The equivalent resonance length is about 2*l*. From mode (ii), the equivalent current on the surface is along one side of the square ring, and the current on the bottom is parallel to the surface, leading to the equivalent resonance length around *a*. From the mode (iii), the equivalent current on the front is the same as the (i), but the current on the bottom is parallel to the front, so the equivalent resonance length is about *a*–*l*. The equivalent resonance length is highest in mode (i), which corresponds to the lowest resonance frequency and the lowest length is in mode (iii), which corresponds to the highest resonance frequency. More detailed information about the principle of polarization conversion is available in Supporting Information Note 1.

### 2.2 The design of radar stealth and digital infrared camouflage compatible metasurface

**Figure 3:**
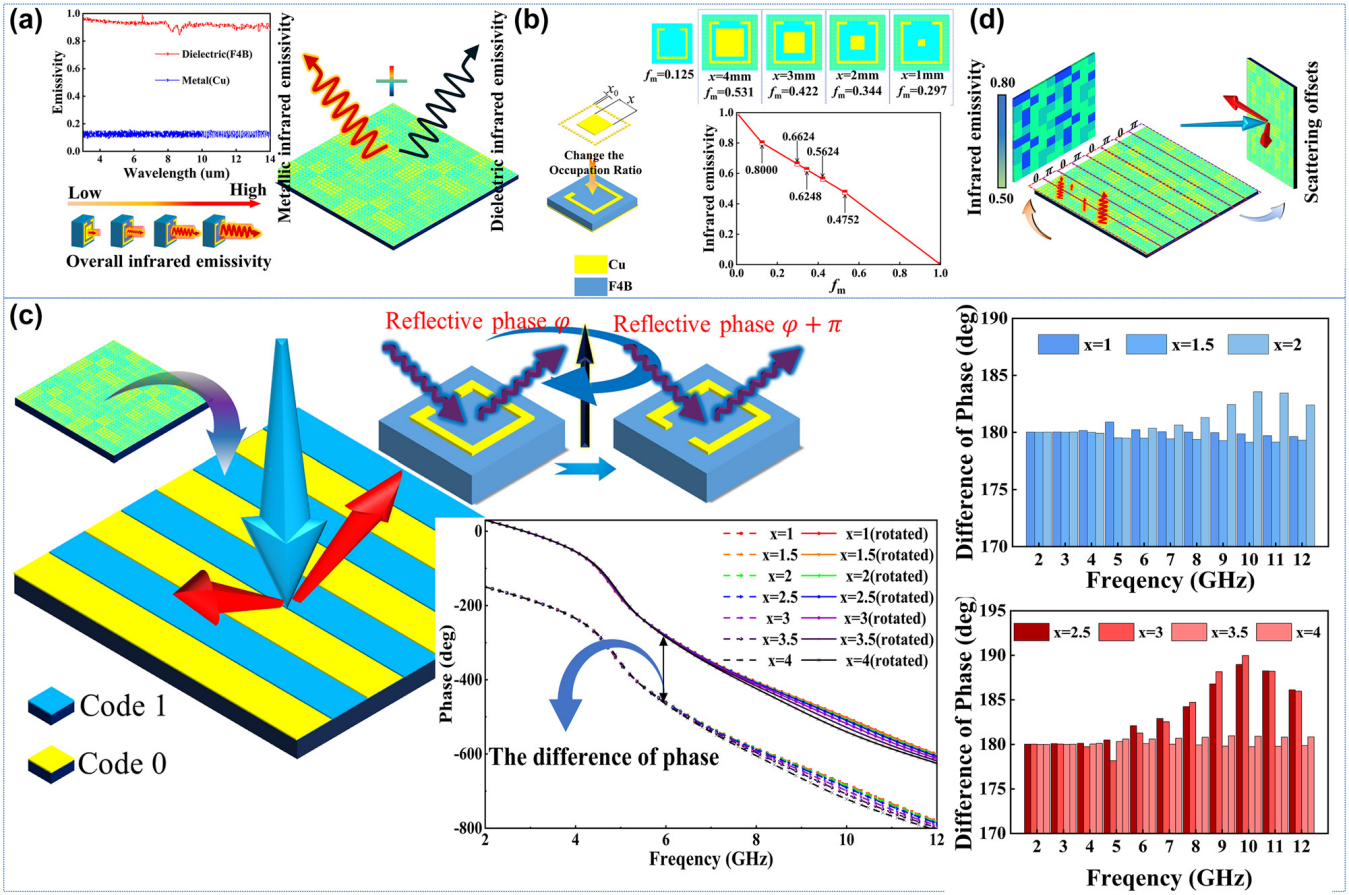
Design and analysis of metasurface with backward RCS reduction and infrared digital camouflage compatibility. (a) The composition of the infrared emissivity and the influence from different components between metal copper and dielectric F4B. (b) The design framework of infrared camouflage with gradient fm. (c) The diagrammatic sketch about the metasurface code 0 and code 1 and the difference phase of meta-atoms code 0 and code 1. (d) The schematic diagram of function for the metasurface with the compatible design of the microwave stealth and infrared camouflage.

Based on the basic rotator in Figure 2(a), the infrared camouflage function is designed by changing the occupation ratio of the meta-atom. Figure 3(a) shows the basic principle of the gradient infrared emissivity. According to the Equation (4) [28]:

(4) $$\varepsilon =\varepsilon_{m}f_{m}+\varepsilon_{d}\left(1-f_{m}\right)$$

where *ε* is the symbol of emissivity, *ε*_*m*_ represents the metal emissivity, *ε*_*d*_ represents the dielectric emissivity, and *f*_*m*_ represents the occupation ratio. Commonly the copper emissivity is 0.1 while the F4B emissivity is 0.9 [29], [30], [31]. According to Equation (4), the infrared emissivity can be changed by designing the occupation ratio. The non-metallic region in the rotator surface provides huge space for changing the occupation ratio. More detailed information about the design method and scheme of the infrared digital camouflage is available in the Supporting Information Note 2. In this way, the design framework of the rotators with the gradient infrared emissivity is shown in Figure 3(b). To make full use of the non-metallic region, the out-ring region is filled by the small metal patches sized *x*_0_ = 0.1 mm, and then the middle region is filled by the metal patch with size from 0 mm to 4 mm, in which the total *f*_*m*_ can cover from 0.125 to 0.531 and the *ε* can be adjusted from 0.8000 to 0.4752 according to the Equation (4).

**Figure 4:**
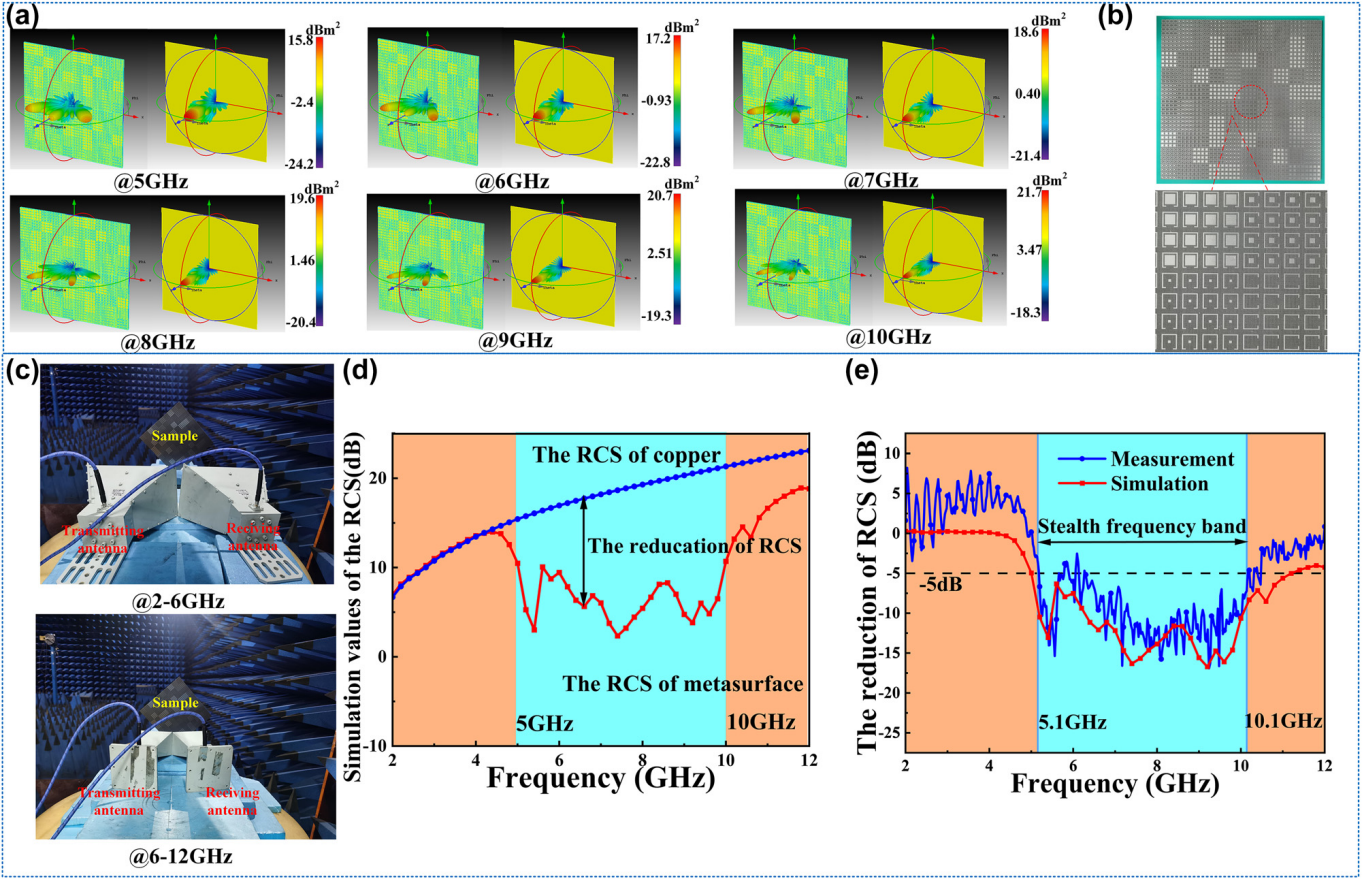
The verification of the microwave performance: (a) the comparison of the far-field simulation results between the metasurface and metal copper at 5 GHz, 6 GHz, 7 GHz, 8 GHz, 9 GHz, and 10 GHz in the 3D impression drawing of the distributions of RCS. (b) The sample of the metasurface. (c) The experimental environment. (d) The RCS comparison of the metasurface and metal with the same size. (e) The comparison of the reduction of RCS between the measurement and simulation.

According to the theory of cross-polarization and Pancharatnam–Berry (PB) phase theory [32], [33], if the rotator is rotated 90°, the phase of the cross-polarization reflective wave will realize a change of 180°. The initializing meta-atom is called code ‘0’, while the rotated meta-atom is called code ‘1’. The alternatively arranged phase coding sequence “00001111…” is introduced to build the coding metasurface, which can scatter the vertical incident electromagnetic waves into two beams, and the reflective waves have deviated, causing a great reduction of the radar cross section (RCS) in the vertical incident [33]. Figure 4(a) shows the diagrammatic sketch of the metasurface code 0 and code 1; with the reflective phase of cross-polarization waves from the code 0 and code 1, the results verify that the 180° difference of phase [34], and the difference is steady from 2 GHz to 12 GHz.

In the guidance of the 0–1 coding theory [35], [36], [37] and P–B phase regulation theory [38], [39], [40], [41], [42], [43], the metasurface with RCS reduction function is designed, at the same time, each rotator on the metasurface is designed by different occupation ratio, which promises the effects of digital infrared camouflage, as Figure 4(b) shows. According to the infrared emissivity Equation (4) and the coding metasurface theory, the compatibility of microwave stealth and digital infrared camouflage is realized. More detailed information about the design method and scheme of the infrared digital camouflage is available in Supporting Information Note 3.

Considering the accuracy limitation of the sample fabrication, the integration of the metasurface in this paper removes the small patches around the ring, just keep the large patches in the middle of the rings. It will cause the minimum infrared emissivity to rise from 0.475 to 0.603. For the microwave, the paper compared the difference between rotators with patches and without patches around the ring, and the results show that the cancellation of the patches around the rings will have little influence on the original microwave performance. What’s more, the paper analyzed the compatibility of the infrared camouflage and radar stealth, which is available in Supporting Information Note 4.

## 3 Verification in simulation and experiment

To verify the radar stealth performance of the metasurface, the far-field simulations for the metasurface and metal copper in the CST are taken. The 3D impression drawing of the distributions of RCS is observed. Six frequency points of 5 GHz, 6 GHz, 7 GHz, 8 GHz, 9 GHz, and 10 GHz are choosed to verify the performance in the band from 5.0 GHz to 10 GHz. The results are shown in Figure 4(a), in which the left is the metasurface and the right is the copper. The incident waves are scattered to two main beams, and the amplitude of the vertical direction is reduced compared with the metal copper. However, there are still side beams on the surface, and the beam in the vertical is not eliminated, which might be caused by three reasons. For the first one, the size of the metasurface is small in the simulation environment, and the wavelength effect from the microwave is obvious. Secondly, the different sizes of the metal patches still produce a tiny influence on the microwave scale, especially on the high frequency. Third, the accuracy of mesh generation from the software is also the reason. Meanwhile, we compare the RCS of the metasurface, and the results are shown in Figure 4(d). In the band from 5 GHz to 10 GHz, the reduction of RCS from the metasurface is obvious, which corresponds to the band with high efficiency of PCR. The function of radar stealth is well verified.

To further verify the compatible performance of metasurface in infrared and microwave, we fabricated a prototype of the metasurface through conventional printed circuit board (PCB) techniques. The detailed photograph of it is illustrated in Figure 4(b), which is the same as the metasurface in the CST. The size of this metasurface is 320 × 320 mm including 1600 meta-atoms.

The microwave experiment measuring system is illustrated in Figure 4(c). The measurements were carried out in a microwave anechoic chamber based on a network analyzer (Agilent E8363B) with two pairs of broadband antenna horns whose frequency bands are 2–6 GHz and 6–12 GHz. To keep the same the simulation settings, the sample was rotated 45° and placed on the detection platform. The center of the transmitting antenna and receiving antenna is placed in line with the normal of the metasurface. In this case, the vertical incidence of the antenna was first aligned to the metal backplane, and the RCS data was normalized for calibration. Then, the antenna was aligned to the sample surface to measure the co-polarization reflection coefficient, and the RCS reduction data of the metasurface was directly obtained, which is shown in Figure 4(e). Compared to the simulation results, the measured results are approximately the same with slight frequency deviation. The difference between the simulation curve and the experimental curve is caused by the following aspects: First is the measuring step size difference between the simulation and experiments. The simulated step size is 0.2 GHz while the experimental step size is 0.03 GHz. Second is the experimental systematic errors, such as the antenna noise interference, the fabrication precision, the finite number of meta-atom cells and the loss of transmission lines. However, the simulation and experimental curves are basically consistent in trend, showing more than 50 % RCS reduction at 5–10 GHz, fully demonstrating the microwave performance of the metasurface.

**Figure 5:**
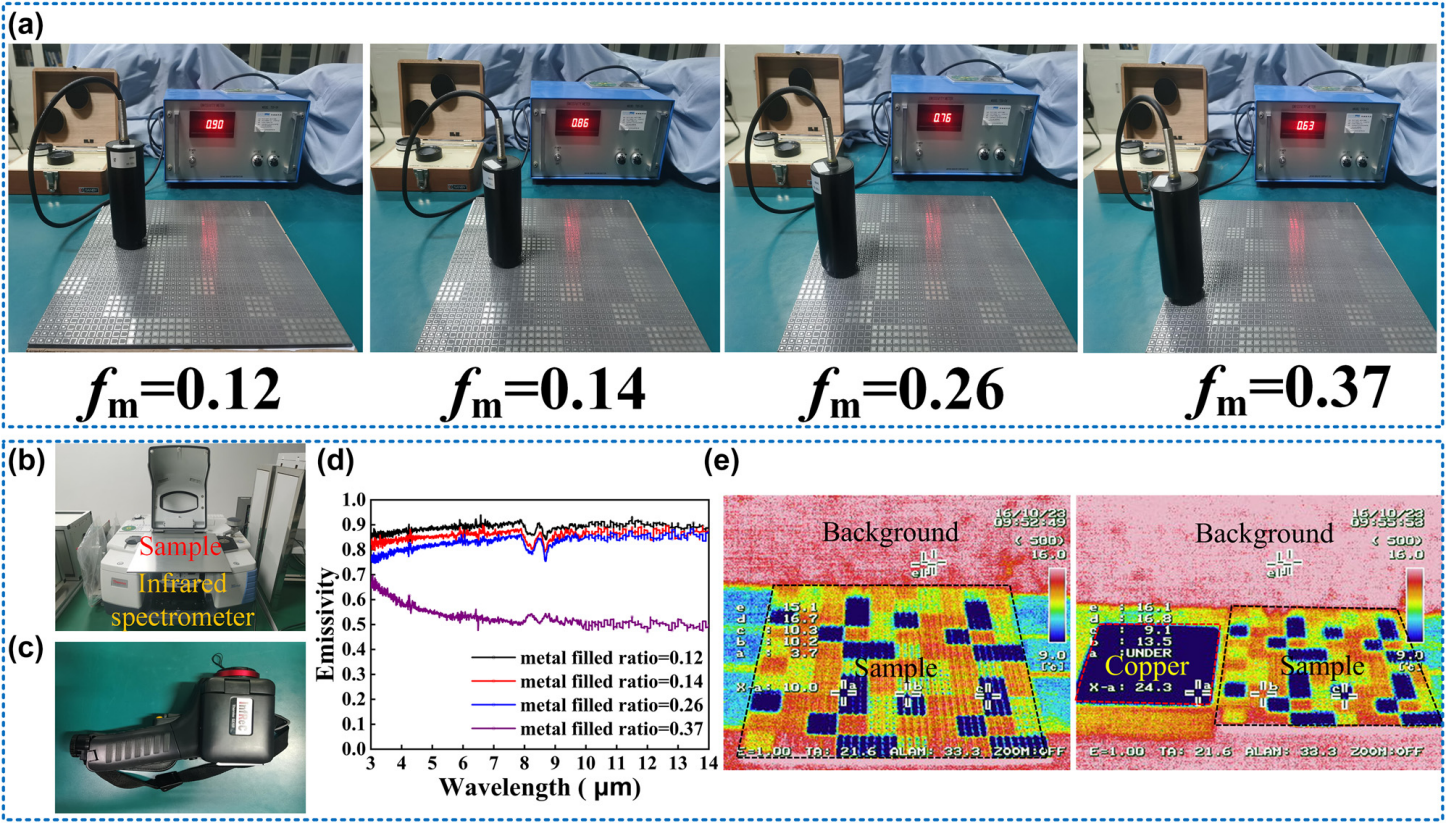
The verification of the infrared performance: (a) mean infrared emissivity measurement in different regions of the sample. (b) The infrared spectrometer. (c) The infrared imager IR. (d) Emissivity spectra in the infrared band at different regions. (e) The overall infrared imaging of the sample and the comparison between the sample and copper.

The infrared emissivity results with different occupation ratios were measured roughly by the TSS-5X IR Emissivity meter first. The infrared probe acts as the detector and the metasurface is fixed horizontally on a platform. An infrared detector was placed on the metasurface to obtain the infrared emissivity in different areas. The results are shown in Figure 5(a), which shows the obvious characteristic of the gradient infrared emissivity and coincides well with the theoretical values. To measure more accurately, we also used an infrared spectrometer instrument to measure the infrared emissivity in the IR range of 3–14 μm, the measured equipment is shown in Figure 5(b). Using a calibrator for calibration, the metasurface was fixed tightly on the instrument to measure the IR emissivity in the areas with different occupation ratios. Due to the large size of the sample, we measured three times and calculated the average values, the results are shown in Figure 5(d), which once again prove the gradient infrared emissivity of the metasurface. There are slight deviations among the data from the TSS-5X IR Emissivity meter and theoretical values, which is possibly caused by the background environment and limitation of instrument precision. What’s more, the roughness of the sample can also have a great influence on the actual infrared emissivity.

**Figure 6:**
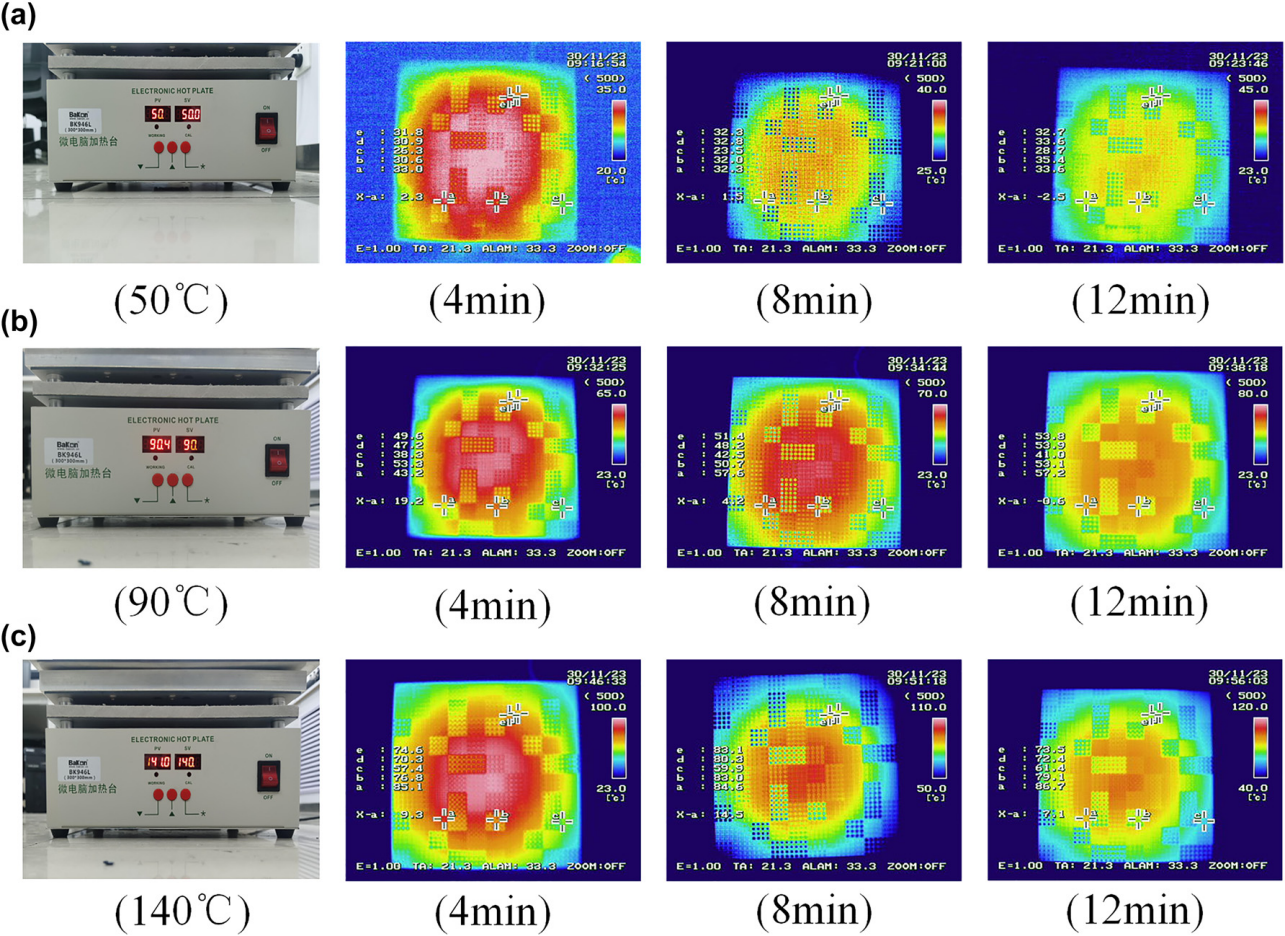
Thermal robustness tests of infrared digital camouflage imaging: (a) the digital camouflage imaging in 50 °C; (b) the digital camouflage imaging in 90 °C; (c) the digital camouflage imaging in 140 °C.

Finally, we used the Infrared imager (Thermao GEAR) to realize the infrared imaging of the sample, which is shown in Figure 5(c). Because of the different infrared emissivity of the surface, the sample has an obvious digital infrared camouflage effect from Figure 5(e), when compared with the metal copper, the effect of camouflage is more obvious.

To further verify the practicality of our designed samples, we tested the infrared digital camouflage performance of the sample at different temperatures to ensure that the samples can adapt to the complex and changeable temperature environment. As the Figure 6(a–c) shows, three temperature gradients of 50 °C, 90 °C, and 140 °C were selected to observe the infrared properties of the samples. At each temperature, the sample is gradually heated over time, which is conducive to the better realization of the camouflage function of the sample. At the same time, the sample still maintains infrared digital camouflage characteristics at different temperatures, indicating that the sample has good thermal robustness and thermal stability, proving that the sample can maintain stable performance in extreme temperature environments (The detailed information about the Thermal robustness tests of infrared digital camouflage imaging can be shown in Supporting Information Note 5).

The experiment results are consistent with the simulation results, which convincingly verified the design of the metasurface has radar stealth and infrared camouflage compatibility performance.

## 4 Conclusions

In this work, we have proposed and designed a single-layer radar stealth and digital infrared camouflage compatible metasurface based on polarization conversion and infrared gradient occupation ratio. First, a polarization rotator is designed to have high efficiency in polarization conversion at 5–10 GHz. Then the multiple plasmon resonances theory is used to analyse mechanism. Based on the rotator, a metal patch is added to the surface of the rotator and changed the occupation ratio to achieve gradient infrared emissivity. At the same time, the change of the infrared emissivity is explained to prove the compatibility with the performance in the microwave band. Then the arrangement of the original meta-patterns is alternated. The meta-patterns of the rotators are rotated to construct coding metasurface to realize the radar stealth. In addition, different occupation ratios are designed for each rotator to achieve the overall digital infrared camouflage effect of the metasurface. Finally, a prototype is fabricated. The experimental results of the sample are consistent with the simulation results, effectively proving the scientificity and reliability of our design. More importantly, only one functional layer of metasurface is used to realize the multi-functions of radar stealth and digital infrared camouflage, which greatly simplifies the complexity of material design, and is of great significance to the large-scale preparation.

## Supplementary Material

Supplementary Material Details
