# Supplementary material for: Noninvasive inset-integrated meta-atom for achieving single-layer metasurface simultaneously with coded microwave reflectivity and digitalized infrared emissivity
Source: Nanophotonics. 2024 May 16;13(17):3113–22. doi: 10.1515/nanoph-2024-0098 (PMC11501719; doi:10.1515/nanoph-2024-0098)
Supplement: Supplementary file 1 — Supplementary Material Details [file j_nanoph-2024-0098_suppl_001.docx]

Supporting Information for

“Noninvasive Inset-integrated Meta-Atom for Achieving Single-Layer Metasurface Simultaneously with Coded Microwave Reflectivity and Digitalized Infrared Emissivity”

Hui-Ting Sun^1,2,3^, Jun Wang^1,2,3*^, Rui-Chao Zhu^1,2,3*^, Zun-Tian Chu^1,2,3^, Xin-Min Fu^1,2,3^, Yu-Xiang Jia^1,2,3^, Yi-Na Cui^1,2,3^, Ya-Juan Han^1,2,3^, Tian-Shuo Qiu^1,2,3^, Sai Sui^1,2,3^, Jia-Fu Wang^1,2,3^*, Shao-Bo Qu^1,2,3^

1 Air Force Engineering University, Xian, China,710051

2 Shaanxi Key Laboratory of Artificially-Structured Functional Materials and Devices, Xian, China, 710051

3 Suzhou Laboratory, Suzhou, China, 215000

Corresponding authors’ E-mail:

*[wangjun563@163.com](file:///D:\科研实践\红外兼容隐身\成果整理\投稿部分\第二版投稿部分\wangjun563@163.com)

*zhuruichao1996@163.com

*[wangjiafu1981@126.com](mailto:wangjiafu1981@126.com)

The supporting information includes:

Note 1. The principle of polarization conversion

Note 2. The design method and scheme of the infrared digital camouflage

Note 3. The design method and scheme of RCS reduction

Note 4. The compatibility analysis of the infrared camouflage and radar stealth

Note 5. The infrared digital camouflage imaging as the heating time increasing

Supplementary Note 1. The principle of polarization conversion

To further understand the mechanism of the polarization conversion, we use the multiple plasmon resonances theory to explain it^1-3^. As shown in Figure 2(a) and (c), the rotator can be essentially conceptualized as a hybrid of a v-directional resonator and a u-directional resonator. The plasmon resonance eigenmodes of the rotator can be stimulated when the electric field aligns with either the v-axis or the u-axis, respectively. To validate it, we analyze plasmon resonance eigenmodes of the rotator via CST as depicted in Figure 2(d). Figure 2(d) gives the co-polarization reflection in the two modes under the normal incidence. It is shown that there are three dips for the co-polarization reflection in two modes. The eigenmodes (i) and (ii) are excited in mode0, while the eigenmode (iii) is excited in the mode1. When y-polarized waves encounter the rotator as displayed in Figure 2(c), three plasmon resonances are instigated since both u and v components concurrently exist. The superposed contributions from two individual orthogonal electric components excite independently the corresponding plasmon resonance eigenmodes^4^.

Since the electric or magnetic resonance of the rotator implies positive and negative effective permittivities (permeabilities), this phenomenon does not require out-of-phase (in-phase) reflection^5-6^. Therefore, to determine whether resonance is electric or magnetic, it is prudent to examine the distribution patterns of surface current. Consequently, surface currents are tracked at each eigen-frequency as depicted in Figure 2(e). Here, it is observed that as in Figure 2(e)(i) and (iii), the U-shaped resonator in the *u*-polarized scenario traces its roots back to the cut-wire resonator; thus, the entire rotator operates like an enlarged cut-wire resonator. Both magnetic and electric resonances emanate from an antisymmetric and symmetric coupling of currents on the increased cut-wire resonator and the ground sheet, as depicted in Figure 2(e)(i) and (iii), respectively. Alternatively, as shown in Figure 2e(ii), only "antisymmetric" modes are excited on the V-shaped resonator in the v-polarized state, rendering the V-shaped resonator similar to a cut-wire resonator. Analogously, magnetic and electrical resonances emerge from anti-parallel and parallel surface currents along the V-shaped resonator and the ground plane, as shown in Figure 2e(ii). In summary, eigenmodes (i) is induced by magnetic resonance, while eigenmode (ii) and (iii) arise from electrical resonances.Accordingly, the three plasmon resonance paths of the rotator are activated by electric and magnetic resonances.

The electric and magnetic resonances can be analyzed qualitatively according to the electrical length between two metallic layers^1, 4^. The operating wavelength of eigenmode (ii) is smaller than the operating wavelength of eigenmode (iii) and eigenmode (iii) is smaller than eigenmode (i). Therefore, the electrical length between the two metallic parts for eigenmodes (i) is larger than eigenmodes (ii) and (iii) is smallest under the same meta-atom cell thickness.

Following this apprehension of the physics of multiple plasmon resonances, the principle of metasurface polarization conversion becomes clear. Take a normally incident *y*-polarized EM wave $E_{i}$as an example. At electric resonance frequencies (ii) and (iii), the electric dipoles are excited with both *x*- and *y*-polarized components, denoted by ***p****_x_* and ***p****_y_*, respectively. $E_{i}$ and ***p****_y_* results in co-polarization reflection, while ***p****_x_* results in cross-polarization reflection as shown in Figure 2(c)(i).

For magnetic resonances^2-3, 5-6^, Figure 2(c)(i) is an outstanding example of an intuitive image of *y*-to-*x* polarization-conversion reflection at resonance 4.9GHz. $\mu_{vv}$ and $\mu_{uu}$ are usually not large in most cases. Thus, EM waves can interact directly with the metal floor, resulting in out-of-phase reflection. However, at magnetic resonance 4.9GHz, $\mu_{uu}$ can become very large to function as a high impedance surface, thereby initiating in-phase reflection, while $\mu_{uu}$ is still not large. $\mu_{vv}$ and $\mu_{uu}$ are different due to the metasurface presenting anisotropic properties. When the *y*-polarized incidence EM wave impinges on the metasurface, it has two orthogonal equal components due to an angle of 45° designed between the *y* and *v* axes, that is, $E_{ui}$=$E_{vi}$. Reflective components $E_{vr}$ $E_{ur}$are generated by out-phase and in-phase reflections at resonance 4.9GHz. That means $E_{vr}$ has a 180 phase difference concerning $E_{vi}$ after reflected by the metasurface, while $E_{ur}$ has the same phase as $E_{ui}$. Hence, the *y*-polarized incident wave is converted to an *x*-polarized reflected wave.

Supplementary Note 2. The design method and scheme of the infrared digital camouflage

Infrared digital camouflage is a new generation of camouflage technology. It uses the visual psychological characteristics of human graphics, digital imaging point matrix characteristics and human visual recognition laws, in a complex infrared background, a high degree of simulation of the background color, texture and other detail information, and in the form of digital "point matrix" presented on the target surface, thereby reducing the distinction between the target and the background, in order to achieve better infrared camouflage effect.

The design principle of infrared digital camouflage is as follows: the same target surface has at least three kinds of infrared emissivity (IR) areas, that is, more than three kinds of infrared color spots; the IR of different areas should have the gradient characteristics; at the same time, the area accounted for by the IR of the intermediate gradient should be more than 50%. Guided by the above principles, this paper designs an infrared digital camouflage surface composed of four gradients of IR of 0.803, 0.790, 0.752, and 0.603, where the proportion of the regions with IR of 0.790 and 0.752 is 50%.

As mentioned in the main text, the design of the IR gradient is mainly realized by controlling the size of the metal patch in the middle of each resonator, and the corresponding metal patch size for each IR is, 1mm*1mm, 2mm*2mm, and 4mm*4mm, respectively. The different sizes of the metal patches indicate that the metal duty cycle of the whole resonator meta-atom is different, and according to Equation 4, the IR is also different. Whether different metal sizes will affect the polarization and rotation performance of the resonator and thus change the microwave performance is discussed in Supplementary Note 2 and Part 2.2 and 2.3.

Considering the actual infrared detection, the distance of the detector from the surface of the target and its own recognition accuracy, the detection type can be categorized into ‘point detection’ and ‘surface detection’. As a long-distance detection method for large targets, ‘surface detection’ has important applications in complex environment camouflage such as mountains and forests. The infrared camouflage designed in this paper is mainly carried out for this means of detection, so each infrared color block in the infrared digital camouflage metasurface designed in this paper is composed of 4*4 resonator meta-atoms of the same IR to ensure that each color block can be picked up by an infrared detector and identified. The specific IR digital camouflage metasurface design is shown in Figure 1(a), and the IR imaging simulation is shown in Figure 1(b).


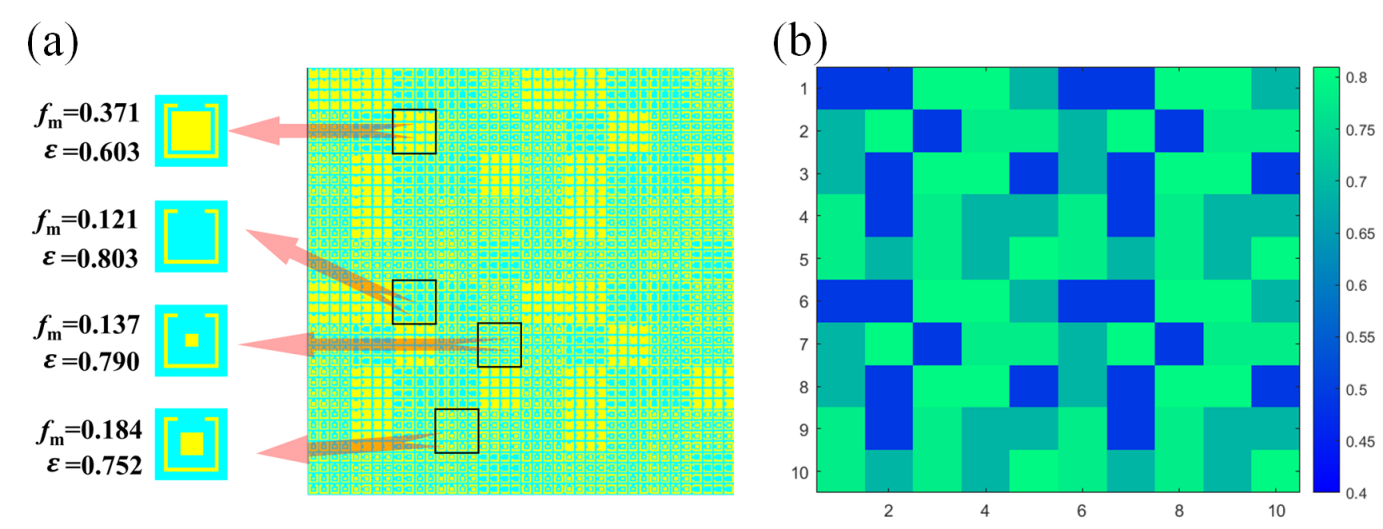


Figure 1. (a) Schematic of the infrared digital camouflage metasurface; (b) metasurface infrared imaging effect

Supplementary Note 3. The design method and scheme of RCS reduction

Means of phase regulation include PB phase, resonance phase, etc. In 2014, researchers introduced the concept of PB phase and investigated its relation to the angle of rotating meta-atoms^7-10^. When the meta-atom rotates around its center by *α*, the phase difference will be 2*α*.

To further analyze the properties of the PB phase, the dipole model is used to decipher. Consider the electric dipole momentum induced by a beam of electromagnetic waves normally incident on the dipole. The electric dipole momentum can be expressed as^8^:

$\left[\begin{matrix} P_{x} \\ P_{y} \end{matrix}\right]=\alpha_{e}\left[\begin{matrix} \cos^{2}\theta& \cos\theta\sin\theta\\ \cos\theta\sin\theta& \sin^{2}\theta\end{matrix}\right]\left[\begin{matrix} E_{x} \\ E_{y} \end{matrix}\right]$ (1)

where *P_x_*, *P_y_*, *E_x_* and *E_y_* are the components of the electric dipole momentum and *α_e_* is the electric polarizability. If the electromagnetic wave is circular polarization, the electric field along *x* and *y* direction has a component, respectively. According to the electromagnetic theory^8-10^, the *P_L(R)_* has such a relationship with *P_x_* and *P_y_*:

$P_{L(R)}=P_{x}\pm jP_{y}$ (2)

where the *L*(+) and *R*(-) represent left- and right- handed circular polarization wave, respectively. According to formula (1), (2), we can get^11^:

$P_{L(R)}=\frac{1}{2}\alpha_{e}(e_{x}+je_{y})\pm\frac{1}{2}\alpha_{e}e^{\pm j2\theta}(e_{x}\mp e_{y})=\frac{1}{\sqrt{2}}\alpha_{e}e_{L(R)}\pm\frac{1}{\sqrt{2}}\alpha_{e}e^{\pm j2\theta}e_{R(L)}$ (3)

where *e_x_* and *e_y_* represent meta-atom vectors along the *x* and *y* directions, respectively, and *e_L_* and *e_R_* represent meta-atom vectors for left- and right-hand circular polarization, respectively. Equation (3) shows that for the incident wave, the reflected wave consists of two components, and the cross-polarized wave is phase-shifted (PB phase) only when the meta-atom rotates around the center. Furthermore, the rotation angle can be adjusted from 0 to *π* so that the phase shift covers 2*π*.

The resonance phase describes the discontinuity produced by the emitted EM waves when the metamaterial interacts with them. It can be explained and analyzed by using the spring oscillator model. The oscillator is subjected to a periodic external force *F* and its dynamical equations are^11-13^:

(4)

where the *ω* is the resonant frequency of the oscillator, *γ* is the spring damping,$F=f\cos(\varpi t)$. We can obtain the oscillator amplitude *c* as follows^11-13^:

$c=\frac{f}{\varpi_{0}^{2}-\varpi^{2}+j\gamma\varpi}=\frac{f}{\sqrt{\left(\varpi_{0}^{2}-\varpi^{2}\right)^{2}+\varpi^{2}\gamma^{2}}}e^{-j\tan^{-1}(\frac{\varpi\gamma}{\varpi_{0}^{2}-\varpi^{2}})}$ (5)

The Phase change of amplitude , by regulating the *ω*_0_, the maximum phase change of 180° can be obtained. By changing the geometric size, the resonant frequency of the cell can be changed to obtain the resonant phase, while the resonant phase of the single-cell system can only cover 180°. In metasurface design, 360° phase coverage is usually achieved by combining PB phases or using complex structural designs with multiphase coupling.

In the case of a paraboloid, reflected waves can be focused because all waves are the same distance from the focus. However, for planar metasurface, the propagation path length of the reflected wave will be different. Therefore, to achieve focusing, the metasurface should compensate for the phase difference generated by different paths. For this purpose, the reflection phase of each element constituting the metasurface can be freely designed. The required phase contribution for each element can be calculated by the equation^7-8, 10, 13^:

(6)

The metasurface consists of $m\times n$meta-atoms. *P* is the meta-atom periodicity. *F* represents the focus length. is the reflected phase of the center element. Figure 2 shows the calculated phase profile required for focusing. The whole metasurface consists of 40×40 meta-atoms, which the periodicity is 5^14-16^. We can obtain the desired phase by adjusting the diagram of the circular patches and arranging them according to the phase profile, as shown in Figure 2.


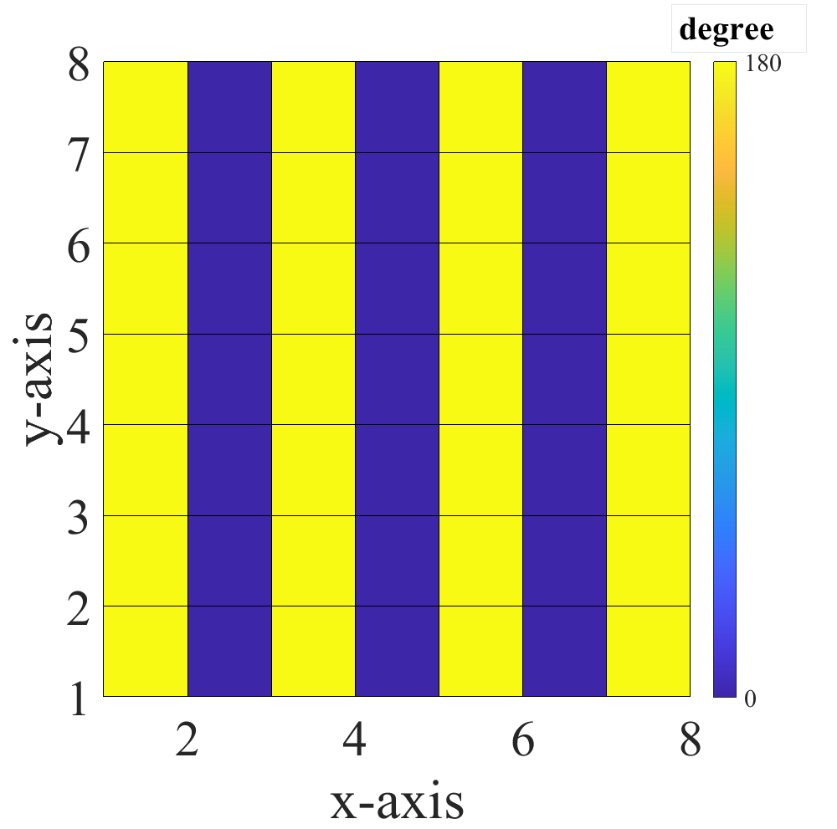


Figure 2. The phase profile of the scattering

Then, we consider manipulating the scattering on the surface of the element by arranging the top layer meta-atom. In the published literature, researchers have done a lot of meaningful work on lowering RCS. One of the most important studies is the proposed alternate structure. Its operation mechanism is based on the counteracting effect of two contributions. If the scattered fields of two meta-atoms have the same amplitude and 180° phase difference, the total scattered field will be cancelled out in the normal direction and turn in two opposite directions.

Supplementary Note 4. The compatibility analysis of the infrared camouflage and radar stealth

The key to demonstrating that compatibility between infrared camouflage and radar stealth functionality can be achieved on a single-layer metasurface is to prove that changing the occupation ratio on the surface of each meta-atom will not affect the polarization conversion performance of the rotator.

Figure.3(a)(i-iv) gives the co-polarization reflectance, cross-polarization reflectance, polarization conversion rate and cross-polarization reflection phase of the rotators with different occupation ratios, respectively, and it can be found that in 5.0-10.0 GHz, the co-polarization reflectance of the rotators with five occupation ratios are all lower than 60%, the cross-polarization reflectance is higher than 80%, and the polarization conversion rate is higher than 63%; and the polarization conversion performance of the rotators with 4.9-9.3 GHz have even better performance, with their co-polarization reflectance below 35%, cross-polarization reflectance above 90%, and polarization conversion rate is above 88%; the cross-polarization reflection phases are basically the same in the range of 5.0-10.0 GHz, which suggests that the microwave performances can all reach the basic indexes under the given gradient of infrared emissivity. Figure 3(b) (i-iv) then analyzes the mechanism in which we observe the surface currents of the rotator with different occupation ratios at 7.0 GHz through a field monitor, and it can be found that when the size of the metal patch is relatively small, the currents excited on the surface of the metal patch is small, which has little effect on the rotator, and thus has little influence on the overall microwave performance of the meta-atom. As the size of the metal patch increases and the distance between the metal patch and the resonator decreases, the surface currents it generates gradually have bigger influence on the rotator, in turn gradually affecting the microwave performance of the meta-atom. It is the reason that the size of the metal patch cannot be infinitely large. At the same time, with the increasing microwave frequency, the oscillation of the current on the surface of the meta-atom is increasing and its stability is weakening, resulting in the increasing current coupling between the metal patch and the rotator, which is the reason why the higher the electromagnetic frequency is, the greater the difference in the microwave performance of the meta-atoms with different occupation ratios. Considering the limitation of the processing accuracy and other reasons, in the process of finalizing and processing the final samples, we chose to remove the small metal patches at the periphery and retain only the metal patches in the middle region, which is a balance between the processing cost control and infrared performance modulation. Figure. 3(c-d) demonstrates that this choice has a minimal effect on the microwave performance, although it has effect on the infrared performance. Figure 3(c) gives the microwave performance curves with and without the peripheral metal patches, and it can be found that the curves in the two cases basically overlap; Figure 3(d) is a precise observation of the difference between the two curves, and the difference between the two cases is magnitude of 0.01, which can be considered to have an extremely small effect.


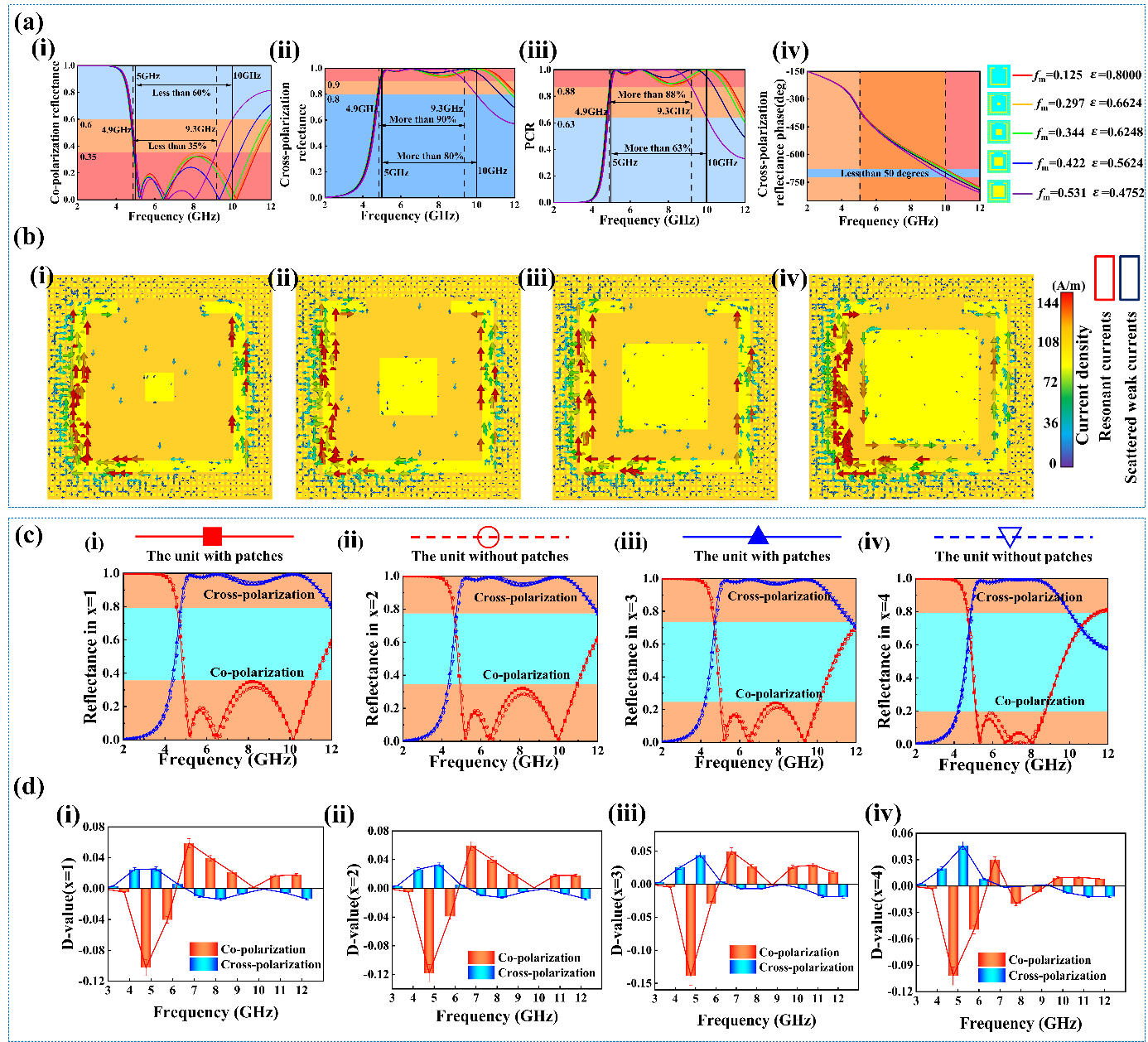


Figure 3. (a)(i-iv) The microwave performance on polarization conversion corresponding to meta-atoms with gradient fm in co-polarization reflectance, cross-polarization reflectance, PCR, and cross-polarization reflective phase, respectively; (b)(i-iv) The surface current distributions on the meta-atoms with different *f_m_* on the 7GHz; (c)(i-iv) The comparison in the polarization performance between the meta-atoms with patches around the ring and without patches around the ring corresponds to x=1, x=2, x=3, and x=4, respectively; (d)(i-iv) The D-values of the polarization performance between the meta-atoms with patches around the ring and without patches around the ring correspond to x=1, x=2, x=3, and x=4, respectively.

Supplementary Note 5. The infrared digital camouflage imaging as the heating time increasing


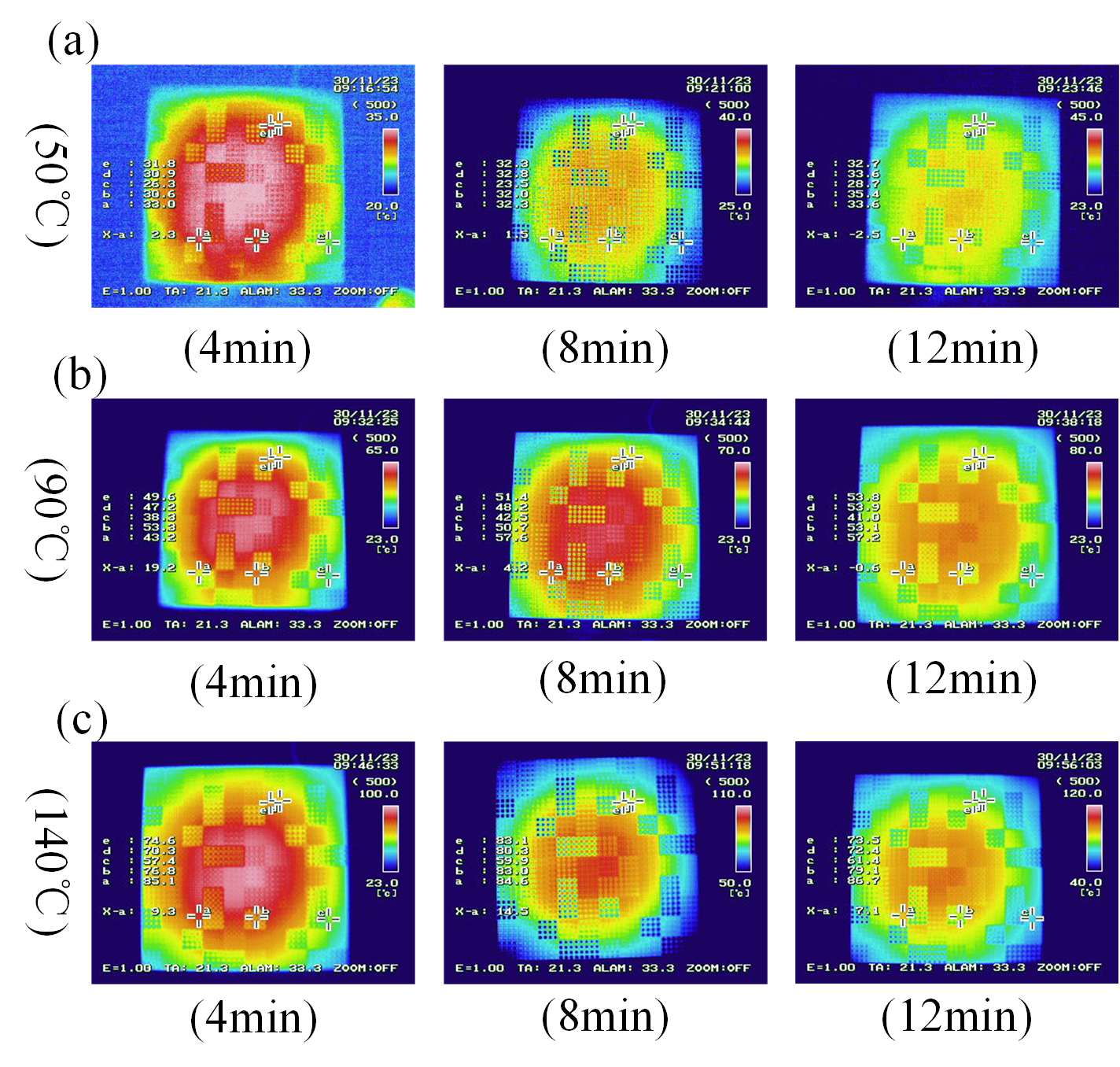


Figure 4. The infrared imaging at different temperatures.

References

1. Chen, H.; Wang, J.; Ma, H.; Qu, S.; Xu, Z.; Zhang, A.; Yan, M.; Li, Y. Ultra-wideband polarization conversion metasurfaces based on multiple plasmon resonances. *Journal of Applied Physics* **2014,** *115* (15), DOI: 10.1063/1.4869917.
2. Cheng, H.; Chen, S.; Yu, P.; Li, J.; Xie, B.; Li, Z.; Tian, J. Dynamically tunable broadband mid-infrared cross polarization converter based on graphene metamaterial. *Applied Physics Letters* **2013,** *103* (22), DOI: 10.1063/1.4833757.
3. Chiang, H.-P.; Lin, J.-L.; Chen, Z.-W. High sensitivity surface plasmon resonance sensor based on phase interrogation at optimal incident wavelengths. *Applied Physics Letters* **2006,** *88* (14), DOI: 10.1063/1.2192622.
4. Huang, X.; Yang, D.; Yang, H. Multiple-band reflective polarization converter using U-shaped metamaterial. *Journal of Applied Physics* **2014,** *115* (10), DOI: 10.1063/1.4868076.
5. Rajkumar, R.; Yogesh, N.; Subramanian, V. Cross polarization converter formed by rotated-arm-square chiral metamaterial. *Journal of Applied Physics* **2013,** *114* (22), DOI: 10.1063/1.4846096.
6. Yang, G.-H.; Liu, X.-X.; Lv, Y.-L.; Fu, J.-H.; Wu, Q.; Gu, X. Broadband polarization-insensitive absorber based on gradient structure metamaterial. *Journal of Applied Physics* **2014,** *115* (17), DOI: 10.1063/1.4868090.
7. Chen, X.; Huang, L.; Muehlenbernd, H.; Li, G.; Bai, B.; Tan, Q.; Jin, G.; Qiu, C.-W.; Zhang, S.; Zentgraf, T. Dual-polarity plasmonic metalens for visible light. *Nature Communications* **2012,** *3*, DOI: 10.1038/ncomms2207.
8. Ding, X.; Monticone, F.; Zhang, K.; Zhang, L.; Gao, D.; Burokur, S. N.; de Lustrac, A.; Wu, Q.; Qiu, C.-W.; Alu, A. Ultrathin Pancharatnam-Berry Metasurface with Maximal Cross-Polarization Efficiency. *Advanced Materials* **2015,** *27* (7), 1195-1200, DOI: 10.1002/adma.201405047.
9. Fan, Y.; Qu, S.-B.; Wang, J.-F.; Zhang, J.-Q.; Feng, M.-D.; Zhang, A.-X. Broadband anomalous reflector based on cross-polarized version phase gradient metasurface. *Acta Physica Sinica* **2015,** *64* (18), DOI: 10.7498/aps.64.184101.
10. Fu, X.; Han, Y.; Fan, Y.; Wang, J.; Zheng, L.; Xu, Z.; Qu, S. FSS-embedded substrates: a facile method of augmenting functions of metasurfaces. *Optics Express* **2021,** *29* (26), 43531-43543, DOI: 10.1364/oe.439179.
11. Ji, R.; Song, K.; Guo, X.; Xie, X.; Zhao, Y.; Jin, C.; Wang, S.; Jiang, C.; Yin, J.; Liu, Y.; Zhai, S.; Zhao, X.; Lu, W. Spin-decoupled metasurface for broadband and pixel-saving polarization rotation and wavefront control. *Optics Express* **2021,** *29* (16), 25720-25730, DOI: 10.1364/oe.431740.
12. Joe, Y. S.; Satanin, A. M.; Kim, C. S. Classical analogy of Fano resonances. *Physica Scripta* **2006,** *74* (2), 259-266, DOI: 10.1088/0031-8949/74/2/020.
13. Yu, P.; Li, J.; Tang, C.; Cheng, H.; Liu, Z.; Li, Z.; Liu, Z.; Gu, C.; Li, J.; Chen, S.; Tian, J. Controllable optical activity with non-chiral plasmonic metasurfaces. *Light-Science & Applications* **2016,** *5*, DOI: 10.1038/lsa.2016.96.
14. Cui, T.-J.; Liu, S.; Li, L.-L. Information entropy of coding metasurface. *Light-Science & Applications* **2016,** *5*, DOI: 10.1038/lsa.2016.172.
15. Cui, T. J.; Liu, S.; Bai, G. D.; Ma, Q. Direct Transmission of Digital Message via Programmable Coding Metasurface. *Research* **2019,** *2019*, DOI: 10.34133/2019/2584509.
16. Li, L.; Cui, T. J.; Ji, W.; Liu, S.; Ding, J.; Wan, X.; Li, Y. B.; Jiang, M.; Qiu, C.-W.; Zhang, S. Electromagnetic reprogrammable coding-metasurface holograms. *Nature Communications* **2017,** *8*, DOI: 10.1038/s41467-017-00164-9.
